# The effect of diurnal light cycles on biohydrogen production in a thermosiphon photobioreactor

**DOI:** 10.1186/s13568-023-01534-x

**Published:** 2023-03-03

**Authors:** Catharine Elizabeth Bosman, Petrie van Wyk, Robert William McClelland Pott, Steven Martin Bradshaw

**Affiliations:** 1 Department of Process Engineering, Stellenbosch University, Banghoek Road, Stellenbosch, 7600 South Africa; 2 Department of Process Engineering, Stellenbosch University, Private Bag X1, Matieland, Stellenbosch, 7602 South Africa

**Keywords:** Diurnal cycle, Illumination, Photobioreactor, *Rhodopseudomonas palustris*, Photofermentation

## Abstract

Hydrogen production via microbial photofermentation shows great promise as a method for sustainable hydrogen production; however, operating costs associated with photofermentative hydrogen production need to be reduced. Costs can be reduced using a passive circulation system like the thermosiphon photobioreactor, and by operating it under natural sunlight. In this study, an automated system was implemented to investigate the effect of diurnal light cycles on the hydrogen productivity and growth of *Rhodopseudomonas palustris* and on the operation of a thermosiphon photobioreactor, under controlled conditions. Diurnal light cycles, simulating daylight times, were found to reduce hydrogen production in the thermosiphon photobioreactor demonstrating a low maximum production rate of 0.015 mol m^−3^ h^−1^ (± 0.002 mol m^−3^ h^−1^) as compared to 0.180 mol m^−3^ h^−1^ (± 0.0003 mol m^−3^ h^−1^) under continuous illumination. Glycerol consumption as well as hydrogen yield also decreased under diurnal light cycles. Nonetheless, hydrogen production in a thermosiphon photobioreactor under outdoor conditions was demonstrated as possible avenue for further investigation.

## Key points

- The thermosiphon photobioreactor can operate under diurnal light cycles.
- Diurnal light cycles reduce hydrogen productivity of *R. palustris*.
- Diurnal light cycles have little to no effect on the growth of *R palustris*.

## Introduction

Purple non-sulphur bacteria (PNSB), such as the species *Rhodopseudomonas palustris*, have the ability to photofermentatively produce hydrogen under anaerobic conditions and in the presence of a suitable carbon substrate. Photofermentative hydrogen has gained increasing attention in the field of green energy; however, questions still exist around the economic feasibility of photofermentation systems. To reduce costs, a novel photobioreactor implementing passive circulation has been proposed—the thermosiphon photobioreactor (TPBR) (Anye Cho and Pott 2019; Bosman et al. 2022a, b). As the name suggests, the TPBR is based on the thermosiphon effect—that is, the reactor utilises a temperature and consequent density differential through heating (as a result of light absorption) on one side and cooling on another side to induce fluid flow and circulation of biomass around the reactor loop (Anye Cho and Pott 2019; Bosman et al. 2022a, b). Another way of reducing associated costs is operating under natural sunlight; therefore, it is important to investigate the effects of factors such as light intensity and illumination periods on photofermentation systems such as the TPBR in order to determine the feasibility of outdoor operation (Uyar et al. 2007).

*R. palustris* cells have been shown to grow and produce hydrogen under a light intensity range of approximately 70–600 W m^−2^ (Ross and Pott 2022); however, there is still some contention in literature with regards to the upper bound at which *R. palustris* can maintain hydrogen activity, with Carlozzi et al*.* reporting hydrogen production at light intensities as high as 803 W m^−2^ (Carlozzi et al. 2010). PNSB have the ability to regulate the number of light harvesting complexes present in their cells, i.e. the number of light harvesting complexes increases under low light intensities in order to fully capture all available photons, and decreases under high light intensities to “avoid photo-damage” (Larimer et al. 2004; Muzziotti et al. 2017). *R. palustris* (strain 420L) has also been reported to exhibit an acclimation strategy when exposed to high light intensities, channelling excess reducing power to the production of molecular hydrogen (Muzziotti et al. 2016). High outdoor light intensities such as that measured in Stellenbosch, South Africa (up to ~ 900 W m^−2^, April 2022) are therefore not expected to adversely affect the bacterial cells. For PNSB, the lower bound on light intensity seems to be the larger issue, as in most cases hydrogen activity ceases in the absence of light (Koku et al. 2002). Nonetheless, during light–dark cycles, hydrogen production has also been shown to resume with illumination, achieving production rates similar to that before the dark period (Koku et al. 2002).

A circadian rhythm in an organism is a property that regulates factors such as metabolic activity with the aim of providing the organism with a fitness/survival advantage (Ma et al. 2016). Circadian organisms generally display three “canonical” characteristics brought about by three “core clock genes” (*kaiA*, *kaiB* and *kaiC*) (Ishiura et al. 1998)—(1) sustained 24-h rhythmicity under constant conditions; (2) a relationship between the “endogenous biological clock” and the diurnal cycle; and, (3) a temperature-compensated rhythmic period, i.e. the period of the biological rhythm remains the same at different temperatures (Ma et al. 2016). *R. palustris* is referred to as a “proto-circadian” organism as the cells do not maintain their rhythmicity under constant conditions and only contain two homologue clock genes [*kaiB*^*Rp*^ (Ma et al. 2016)/*rpa0008* (Larimer et al. 2004) and *kaiC*^*Rp*^ (Ma et al. 2016)/*rpa0009* (Larimer et al. 2004)]. Nonetheless, these cells still exhibit a time-keeping mechanism—when exposed to a light–dark cycle, the photosynthetic activity is governed not only by the presence and absence of light, but also by a 24-h periodicity where the cells seem to anticipate light/dark periods (Ma et al. 2016). Although “proto-circadian”, it is still unclear whether diurnal light cycles could be advantageous to *R. palustris* in terms of growth and hydrogen productivity.

Illumination protocol (the amount of time spent in the light and dark) has been shown to be an important parameter in the optimization of hydrogen productivity by photofermentative bacteria (Miyake et al. 1999; Uyar et al. 2007; Androga et al. 2011). Moving in the direction of outdoor operation for the reduction of costs, several studies have investigated photofermentation and growth of PNSB under outdoor conditions (Kim et al. 1982; Carlozzi and Sacchi 2001; Carlozzi et al. 2006; Androga et al. 2011; Adessi et al. 2012; Boran et al. 2012). However, due to the influence of other varying conditions (temperature, weather, etc.), studying the effects of diurnal light cycles can become rather complex under outdoor conditions. For this reason, several studies have been conducted on the effects of light cycles under controlled indoor conditions (Miyake et al. 1999; Uyar et al. 2007; Androga et al. 2011). To do this, it is imperative to simulate these light cycles as closely as possible; however, the majority of studies implement instantaneous light–dark protocols, i.e. a specified period under constant illumination and a specified period under no illumination (e.g*.* 12 h full light intensity, 12 h complete dark) (Miyake et al. 1999; Uyar et al. 2007; Eroǧlu et al. 2010; Montiel Corona et al. 2017), and step-wise light cycles, i.e. ramping the light intensity up and down in a step-wise manner at specified time-intervals (e.g*.* 1 h at 100 W m^−2^, 1 h at 200 W m^−2^, 1 h at 300 W m^−2^ and so forth, and then down again, approximating daylight light variation) (Miyake et al. 1999). Furthermore, no literature exists on the behaviour of the TPBR when operated under outdoor conditions, or conditions of variable illumination—crucial information necessary for evaluating feasibility of outdoor operation.

This study investigates the effect of diurnal light cycles mimicking natural outdoor conditions, on the growth and hydrogen productivity of the bacterial species *R. palustris*, and on the working of a thermosiphon photobioreactor. Natural diurnal light cycles are closely mimicked with a light simulation system, presenting a light protocol that can also be used in other photofermentation studies.

## Materials and methods

### *B*acterial species and culturing

In this study, *Rhodopseudomonas palustris* (NCIMB 1774) was used as model organism. Cells were precultured in Van Niels medium—a fast-growing medium containing (per 1 L): 1 g K_2_HPO_4_, 0.5 g MgSO_4_, 10 g yeast extract and the balance deionised water (du Toit and Pott 2021). After autoclaving (121 °C, 20 min), 10 mL of sterile glycerol (4 M) was added to the medium aseptically (du Toit and Pott 2021). Bacterial cells were suspended in the medium and grown anaerobically in 500 mL Schott bottles under argon atmosphere. Temperature was maintained at 35 °C (± 0.2 °C) and light intensity was calibrated to 200 W m^−2^ (± 20 W m^−2^) in the wavelength range of 500–1100 nm (tungsten filament incandescent light, Eurolux^©^, South Africa) (Bosman et al. 2022a) using a handheld spectrophotometer (RGB Photonics, Qmini VIS–NIR). Culturing time was approximately five days to allow cells to reach the mid-logarithmic phase.

All growth and hydrogen production experiments were conducted using a *Rhodospirillaceae* medium containing (per 1 L): 0.6 g K_2_HPO_4_, 1.7 g KH_2_PO_4_, 0.02 g MgSO_4_·7H_2_O, 0.005 g CaCl_2_·2H_2_O, 0.4 g NaCl, 0.3 g Na_2_S_2_O_3_, 0.0005 g ferric citrate, 0.0002 g para-aminobenzoic acid, and 1 mL of trace element solution containing (per 1 L): 70 mg ZnCl_2_, 100 mg MnCl_2_·4H_2_O, 60 mg H_3_BO_3_, 200 mg CoCl_2_·6H_2_O, 20 mg CuCl_2_·2H_2_O, 20 mg NiCl_2_·6H_2_O, and 40 mg NaMoO_4_·2H_2_O (Pott et al. 2014). The medium was autoclaved (121 °C, 20 min) and the pH measured at 7.2. Also aseptically added to the medium after autoclaving was a vitamin solution containing (per 1 L): 1.2 g thiamine HCl and 0.01 g cyanocobalamin (filter-sterilized), and lastly 10 mL of 5 M sterile glycerol (final concentration of 50 mM) and 5 mL of sterile glutamic acid (final concentration of 10 mM) were added to the medium aseptically (Pott et al. 2014).

### Experimental setups

For the hydrogen production and growth experiments, two different photobioreactor setups were implemented. A test-tube reactor was used to investigate the effect of light cycles on the growth and hydrogen productivity of the bacterial species *R. palustris* at constant temperatures, while a TPBR setup was used to determine the effect of light cycles on the hydrogen productivity and internal working of the TPBR itself at varying temperatures.

### Test-tube photobioreactor

The test-tube reactor system consisted of four glass test-tubes (72 mL working volume) mounted inside a temperature-controlled water bath (35 °C ± 0.2 °C). The test-tubes were illuminated from one side using halogen floodlights (Eurolux^©^, South Africa, FS13, 150 W) and the light intensity inside the test-tubes was calibrated using a handheld spectrophotometer (RGB Photonics, Qmini VIS–NIR). The tubes were fitted with gastight lids consisting of two stainless steel sample ports—one for liquid samples (biomass and glycerol concentration) and one for gas measurements. The gas port was connected to an inverted measuring cylinder, immersed in a water bath, using low hydrogen-permeability tubing (Saint Gobain, South Africa, Tygon E3606). A one-way valve was also used to ensure no reverse flow into the reactor itself. The water-displacement technique was used to monitor the volume of evolved gas.

### Thermosiphon photobioreactor

The TPBR is a tubular loop (inner diameter 24 mm) reactor made of glass and can be divided into three sections—an illuminated riser section (600 mm length), an insulated downcomer section (450 mm length) and a cooling section surrounded by a cooling water jacket (442 mL volume) (Bosman et al. 2022a). The entire reactor was insulated and shaded from light, except for the illuminated riser section which was illuminated by halogen floodlights (Eurolux^©^, South Africa, FS13, 150 W). A water chiller (model F25, Julabo GmbH, Germany) was used to continuously circulate cooling water through the jacket at a fixed flow rate of 0.5 L min^−1^. The reactor had a reflector in the centre of the loop behind the riser section, facing the riser and the halogen floodlights—this was to ensure light from both sides of the riser section for enhanced light distribution. The reactor was fitted with three temperature sensors (3-wire PT100, 3 mm diameter, stainless steel sheath) that were connected to a data logger—the three sensors were positioned at the top and bottom of the illuminated riser section, and one directly below the cooling section of the TPBR. The reactor was fitted with a GL45 polypropylene lid, and samples were taken from the sampling ports. The volume of evolved gas was quantified using the water-displacement method, similar to that described for the test-tube reactors.

### Illumination system

**Fig. 1:**
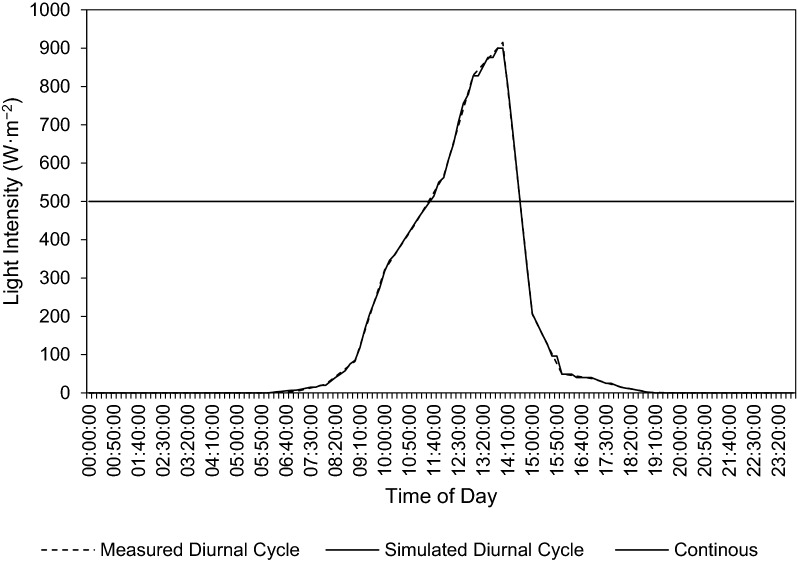
Measured and simulated 24-h diurnal light cycle, and continuous light intensity (500 W m^−2^) over time

In order to simulate outdoor conditions, more specifically the diurnal light cycle, outdoor light intensities were measured. Light intensities within the spectral range of 360–1120 nm were measured using a pyranometer (Campbell Scientific, South Africa, CS300, Apogee Instruments Inc.) connected to a data logger (Campbell Scientific, South Africa, CR300). The light intensities were measured over a period of 24 h (Stellenbosch, South Africa, April 2022) and the mean of triplicate measurements were used. The time-dependent light intensity data were coded into an Arduino^®^ microcontroller light simulator system. The system comprised a microcontroller board (Micro Robotics^©^, South Africa, Arduino^®^ UNO R3 Original) connected to four vertically oriented halogen floodlights (Eurolux^©^, South Africa, FS13, 150 W), a 4-channel AC dimmer module (Rocket Controller, China, 3.3 V/5 V logic, AC 50/60 Hz) and a real-time clock module (Micro Robotics^©^, South Africa, DS3231, 3.3 V/5 V) to maintain accurate time-keeping in the event of a power outage. This allowed automated control of the emitted light intensities, simulating an indoor light cycle mimicking that measured outside (Fig. 1).

### Experimental procedure

For the growth and hydrogen production experiments, precultured *R. palustris* cells were added to *Rhodospirillaceae* medium (with glycerol as carbon source) to obtain a starting biomass concentration of approximately 0.1 g L^−1^ in all reactors. Thereafter, the cultures were aseptically added to an autoclaved (121 °C, 20 min) photobioreactor, i.e. either the test-tube or TPBR setup, depending on the experiments. Reactors were sparged with filtered-sterilised (Midisart^®^, 2000 PTFE filter, diameter of 50 mm, pore size of 0.2 µm) argon gas (purity of > 99.9%) for approximately 10 min to ensure an anaerobic atmosphere inside the reactors.

Experimental runs using the test-tube reactors were initialized by switching on the light sources and the water baths (35 °C). In the TPBR, experimental runs were initialized by switching on the light sources and the cooling system to induce circulation in the reactor. Sampling (cell growth and glycerol concentration) was done approximately every 10, 14 or 24 h, and the cumulative volume of evolved gas was also noted at these timestamps. All test-tube experiments were done in quadruple, while the TPBR runs were conducted in triplicate to allow for the determination of statistical significance and standard deviation—that is, each individual experimental run was repeated four and three times, respectively, at identical conditions.

### Analytical methods

Cell dry weight (CDW) was determined using a CDW *vs* optical density (OD) standard curve. A UV/Vis-spectrophotometer (Model AE-S60-4U) was used to measure OD, and OD was correlated to CDW as follows: CDW = 0.7126 × OD_660 nm_—0.007 (Van Niels medium), *R*^*2*^ = 0.9981; CDW = 0.6391 × OD_660 nm_ + 0.0619 (*Rhodospirillaceae* medium), *R*^*2*^ = 0.9996 (Bosman et al. 2022a). Glycerol concentration was measured using high-performance liquid chromatography (Dionex UltiMate 3000 HPLC). Filtered samples (FilterBio^®^ Nylon Syringe Filter, 13 mm diameter, 0.22 µm pore size) were injected into the HPLC column (Bio-Rad Laboratories Ltd., Johannesburg, South Africa, HPX-87H column, 250 × 7.8 mm with guard cartridge and ERC Refracto Max520 RI detector) operating at 65 °C and using a 0.005 M H_2_SO_4_ solution as mobile phase (0.6 mL min^−1^). Evolved gas samples were analysed using a gas chromatograph (Global Analyser Solutions Compact Gas GC) with a thermal conductivity detector (110 °C), packed columns (Rt-QBond 3 m × 0.32 mm and Molsieve 5A 3 m × 0.533 mm) and argon as carrier gas (45 kPa, 50 µL injections with a split of 5 mL min^−1^). The GC oven and filament temperatures were specified as 65 and 210 °C respectively, and the reference flow rate was 1 mL min^−1^.

### Calculations

The effect of light protocol was investigated based on the rate of hydrogen production, glycerol consumption and hydrogen yield. The rate of hydrogen production was calculated using the molar volume of hydrogen produced (Δ*n*) at the time of interest (*t*) together with the culture/photobioreactor volume (*V*), as denoted in Eq. 1. The molar volume of hydrogen was determined at NTP using the analysed composition of the evolved gas, i.e. ~ 92% (± 1.9%) hydrogen and the balance CO_2_. Glycerol consumption was evaluated using the molar concentration of glycerol in the system at a specific time (*n*_*t*_), and the glycerol concentration initially in the system (*n*_*o*_), as shown in Eq. 2.

1 $$ {\text{Rate of H}}_2 \,{\text{production}}\,\left( {{\text{mol}}\,{\text{m}}^{ - 3} \,{\text{h}}^{ - 1} } \right) \, = \frac{{\Delta n_{{\text{H}}_2 \,measured} }}{Vt} $$

2 $$ {\text{Glycerol consumed}}\,\left( \% \right) = \frac{{n_{o,glycerol} - n_{t,glycerol} }}{{n_{o,glycerol} }} \times 100 $$

Hydrogen yield was determined as the molar ratio of hydrogen produced to the theoretical maximum of glycerol that can be consumed according to the stoichiometric conversion of glycerol to hydrogen: C_3_H_8_O_3_ + 3H_2_O → 3CO_2_ + 7H_2_ (Eq. 3).

3 $$ {\text{H}}_2 \,{\text{yield}}\,\left( \% \right) \, = \frac{{\Delta n_{{\text{H}}_2 \,measured} }}{{7\Delta n_{glycerol\, consumed} }} \times 100. $$

## Results

### Effect of illumination protocol on *R. palustris*

**Fig. 2:**
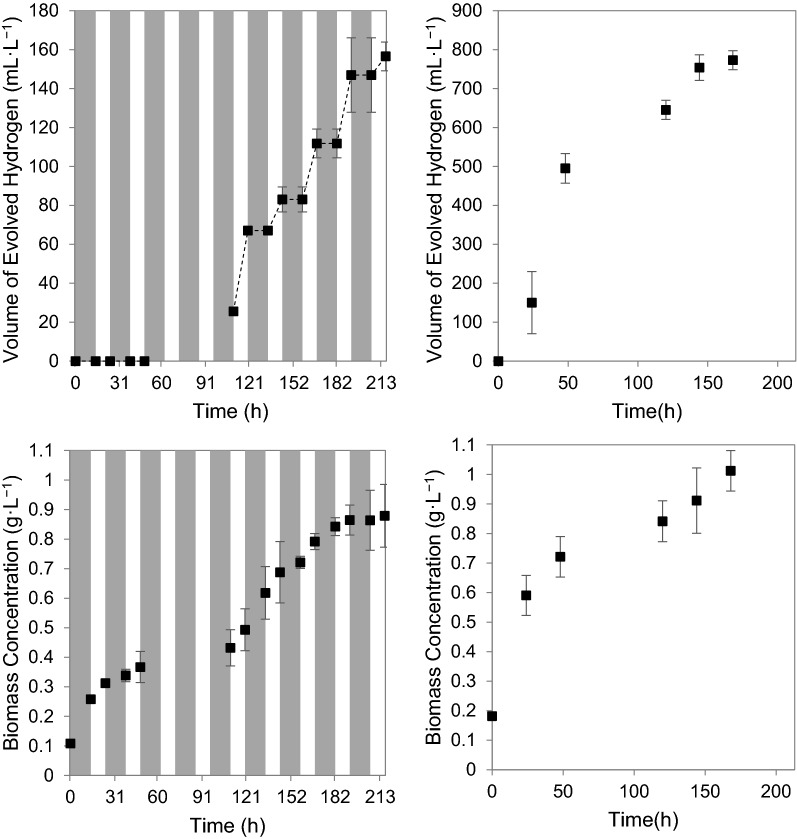
(Top) Cumulative volume of evolved hydrogen gas per test-tube reactor working volume under (left) diurnal light cycles, and, (right) constant illumination at ~ 500 W m^−2^ (± 20 W m^−2^); and, (Bottom) *R. palustris* concentration per test-tube reactor working volume under (left) diurnal light cycles, and, (right) continuous illumination at ~ 500 W m^−2^ (± 20 W m^−2^)*—*dark periods indicated with grey areas; each data point represents the mean of triplicates with the error indicated as standard deviation

Variation in temperature significantly influence the metabolic activities of *R. palustris* (du Toit and Pott 2021). For this reason, the hydrogen production and growth of *R. palustris* under diurnal light cycles were first investigated in the test-tube PBR setup operating under a constant temperature of 35 °C (Fig. 2). This allowed for the investigation of the individual effects of the diurnal light cycle on the photosynthetic activity of *R. palustris*, without the interference of varying temperatures and the effects of biomass circulation, as would be the case in the TPBR. Overall hydrogen production rates of 0.032 mol m^−3^ h^−1^ (± 0.001 mol m^−3^ h^−1^; 216 h) and 0.205 mol m^−3^ h^−1^ (± 0.007 mol m^−3^ h^−1^; 168 h) were demonstrated in the test-tube PBR system under diurnal light cycles and constant light (~ 500 W m^−2^), respectively. Maximum hydrogen production rates of 0.066 mol m^−3^ h^−1^ ± 0.004 mol m^−3^ h^−1^ (between 110 and 192 h) and 0.461 mol m^−3^ h^−1^ ± 0.035 mol m^−3^ h^−1^ (between 0 and 48 h) were achieved under diurnal light cycles and continuous illumination, respectively. The rate of hydrogen production achieved under diurnal light cycles were substantially slower than under constant illumination—this suggests that varying light intensities and dark periods overnight significantly reduce the hydrogen productivity of *R. palustris* even under constant temperatures. During dark periods, hydrogen production ceased completely, and furthermore, the system under diurnal light cycles also displayed a much longer hydrogen production lag phase at the start of the experimental runs (Fig. 2). After 168 h, 45.9% (± 1.8%) of the glycerol had been consumed by the *R. palustris* cells under constant light, while a slightly lower portion of 37.7% (± 2.0%) of the glycerol had been consumed under diurnal light cycles. However, the hydrogen yields were determined to be 3.6% (± 0.7%) and 20.1% (± 1.3%) under diurnal light cycles and constant light, respectively. The substantially lower hydrogen yield under diurnal light cycles for a similar percentage of carbon substrate utilized suggests that a much larger portion of the glycerol substrate was being diverted elsewhere and/or used for the production of other products, e.g*.* potentially poly-hydroxybutyrate (PHB) granules.

In terms of microbial growth, overall growth rates of 0.005 g L^−1^ h^−1^ (± 0.0003 g L^−1^ h^−1^) and 0.006 g L^−1^ h^−1^ (± 0.0004 g L^−1^ h^−1^) were achieved under diurnal light cycles and under constant light, respectively—relatively similar growth rates. The effect of the varying light intensities seemed to be less pronounced on biomass concentration than on hydrogen production, with biomass concentrations still increasing overnight during dark periods (Fig. 2). This suggests that the organism was likely operating partially chemoheterotrophically during the dark periods, utilizing the glycerol substrate as energy source in the absence of light. In addition to the potential production of PHB, this also explains the low hydrogen yield under diurnal cycles for a relatively similar amount of glycerol substrate consumed under constant and diurnal light. When comparing microbial growth under diurnal light cycles and constant light, the main difference seemed to be the initial rate of increase in biomass concentration—after 48 h of operation, a biomass concentration of 0.721 g L^−1^ (± 0.069 g L^−1^) had already been achieved under constant illumination whereas it took approximately 158 h for the system under diurnal light cycles to reach that same biomass concentration of 0.721 g L^−1^ (± 0.030 g L^−1^).

### Effect of illumination protocol on a thermosiphon photobioreactor

**Fig. 3:**
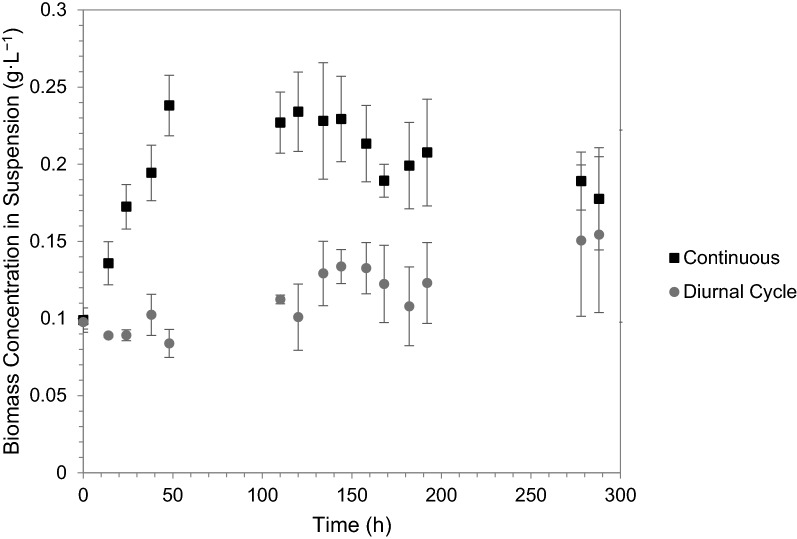
Concentration of R. *palustris* biomass in suspension in the TPBR under continuous halogen illumination at approximately 500 W m^−2^ (± 20 W m^−2^) and under a diurnal illumination cycle*—*each data point represents the mean of triplicates with the error indicated as standard deviation

The experimental work in the test-tube photobioreactor setup allowed for the investigation of diurnal light cycles on *R. palustris* under controlled conditions, i.e. at a constant temperature of 35 °C—thus, the individual effects of diurnal light cycles. In the TPBR, however, diurnal light cycles not only affect the microorganism, but also circulation and biomass suspension in the reactor, temperature profiles as well as nutrient transfer and could have confounding effects on the system as a whole. Investigation of hydrogen productivity and growth under diurnal cycles in the two different systems, therefore, provided qualitative insight into the additional effects of circulation and temperature fluctuations in the TPBR, as a consequence of diurnal light cycles. To investigate the effect of light cycles on the internal working and biomass circulation in a TPBR to determine feasibility of outdoor operation, the reactor’s ability to maintain *R. palustris* cells in suspension was compared under continuous illumination and diurnal light cycles. After 228 h, the concentration of biomass in suspension in the TPBR under continuous illumination (500 W m^−2^ ± 20 W m^−2^) and under diurnal cycle illumination was approximately 0.178 g L^−1^ (± 0.033 g L^−1^) and 0.154 g L^−1^ (± 0.050 g L^−1^), respectively. During dark periods in the diurnal light cycle, circulation in the TPBR ceases as a result of the temperature differential in the reactor dissipating (no light/heat). It is therefore expected that less biomass will be in suspension under diurnal light cycles as some of the biomass will settle out during the dark periods and will not resuspend when illumination resumes. Under continuous illumination, the concentration of biomass in suspension increased substantially in the first 48 h, after which it remained relatively constant. Under light cycles, however, no definitive increase was exhibited with the concentration of suspended biomass remaining relatively constant throughout the duration of the experimental run (Fig. 3). From visual observation, it was noted that some of the *R. palustris* biomass settled out over time—under diurnal light cycles slightly more so than under continuous illumination; therefore, collectively this suggests that the biomass that remained in suspension consisted predominantly of motile ‘daughter cells’, with the larger ‘mother cells’ settling to the bottom (Whittenbury and McLee 1967).

Under continuous illumination, an overall hydrogen production rate of 0.087 mol m^−3^ h^−1^ (± 0.014 mol m^−3^ h^−1^) was achieved after 228 h of operation with the maximum hydrogen production rate exhibited between 48 and 120 h—0.180 mol m^−3^ h^−1^ (± 0.0003 mol m^−3^ h^−1^). Over a period of 228 h, 33.9% (± 0.8%) of the glycerol initially in the system was consumed by the *R. palustris* cells with a glycerol to hydrogen yield of 19.1% (± 2.9%) of the stoichiometric maximum. The maximum hydrogen yield was determined to be 27% (± 8.8%) after a period of 110 h. The hydrogen production lag phase was about 24 h, with hydrogen production activity starting to plateau after 168 h—that is, a hydrogen production window of approximately 142 h.

**Fig. 4:**
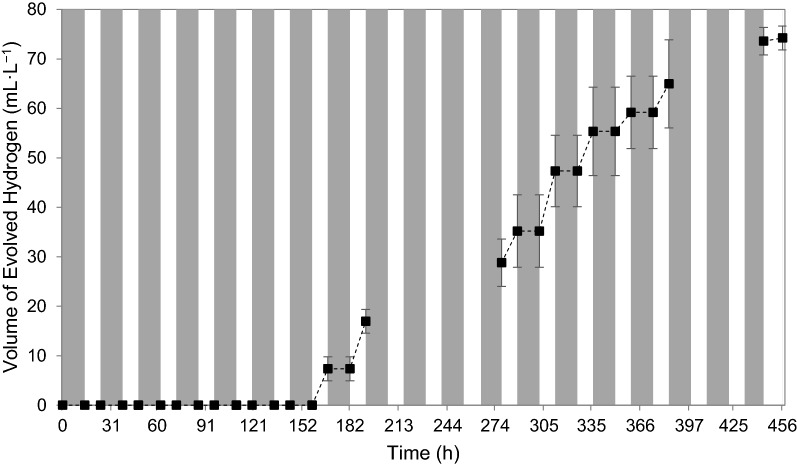
Cumulative volume of evolved hydrogen gas per TPBR working volume (dark periods indicated with grey areas)*—*each data point represents the mean of triplicates with the error indicated as standard deviation

When exposed to varying diurnal cycle light intensities, a hydrogen production lag phase of 158 h was exhibited (Fig. 4)—134 h longer than for a continuous illumination system. Here, hydrogen production activity started to cease after approximately 444 h, which gives a hydrogen production window of about 286 h—twofold that of the continuous illumination system. Considering the light–dark cycles and the period of time under illumination, this was to be expected as the microorganisms were exposed to light only half of the time compared to the continuous illumination system.

Under simulated diurnal light cycles, an overall hydrogen production rate of 0.007 mol m^−3^ h^−1^ (± 0.0002 mol m^−3^ h^−1^) was achieved after 456 h, with a maximum hydrogen production rate of 0.015 mol m^−3^ h^−1^ (± 0.002 mol m^−3^ h^−1^) between 278 and 384 h—rates less than 10% of that achieved under continuous illumination in the TPBR.

**Fig. 5:**
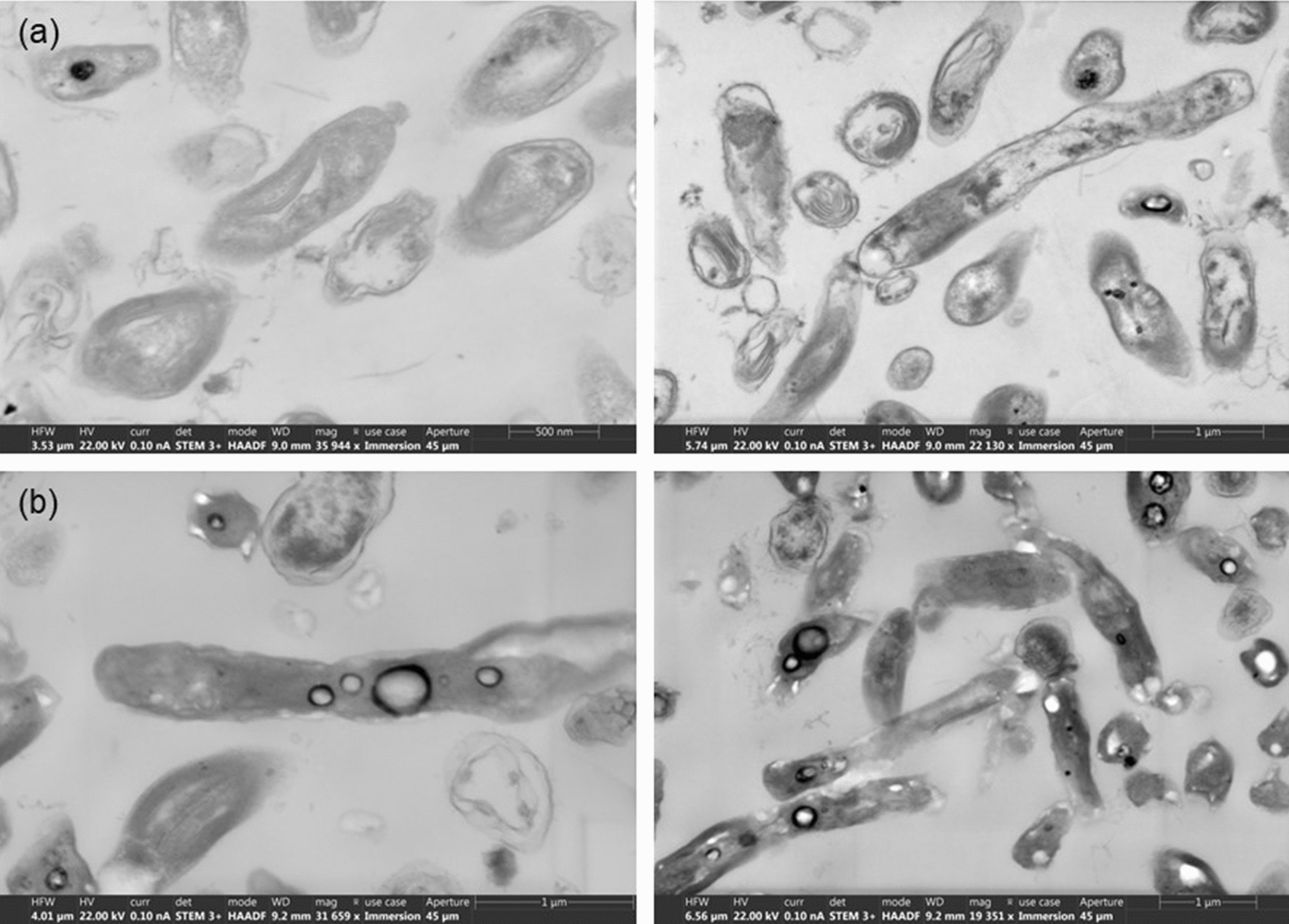
Scanning transmission electron microscopy (STEM) images of R. *palustris* cells **a** before (at 0 h, after preculturing in Van Niels medium); and **b** after (at 456 h) being exposed to a diurnal illumination cycle*—*images taken by Stellenbosch University’s Central Analytical Facilities (CAF)

Due to the TPBR’s dependence on a light/heating source (in order to create the thermosiphon effect), hydrogen production was influenced not solely by the presence/absence of light but also by the TPBR’s ability to restart circulation after dark periods. That is, achieving a sufficient temperature differential in the reactor able to facilitate mixing, nutrient transfer to the organisms and also to resuspend the *R. palustris* cells in the event of them settling out. Furthermore, as *R. palustris* is highly temperature-dependent (du Toit and Pott 2021), the variation in temperature as a result of the light cycles likely also had an effect on the overall hydrogen productivity of the system and the organism’s metabolic activities; however, from the data obtained under constant as well as varying temperatures, it was shown that the varying temperatures in the TPBR system had a slightly less pronounced effect on the hydrogen productivity as compared to the light protocol. Over the course of the experimental run, 24.1% (± 4.0%) of the glycerol initially in the system was consumed by *R. palustris* with a very low hydrogen yield of 3.6% (± 0.7%). This suggests diversion of glycerol reducing equivalents to products other than the desired hydrogen gas, i.e. internal storage products such as PHB, similar to the test-tube reactor setup. The presence of PHB granules was investigated qualitatively through scanning transmission electron microscopy (STEM) images, where white granules were observed in the bacterial cells after being exposed to 456 h of varying diurnal light cycles (Fig. 5). These white granules were not visually observed at the start of the experimental runs, suggesting production of PHB during the course of the diurnal light experiments (Fig. 5). Some PHB granules were also produced under continuous illumination, but comparably less than under diurnal light cycles.

**Fig. 6:**
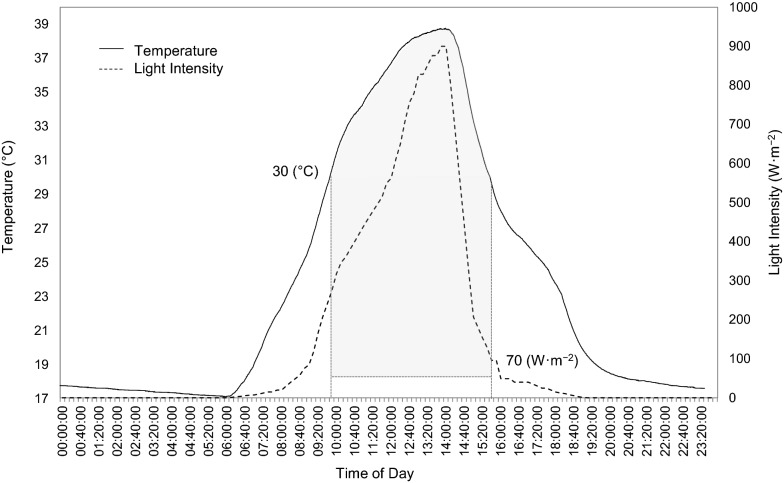
Operating temperature in the TPBR under indicated diurnal light cycles (hydrogen production range indicated by shaded area)

Under continuous illumination of ~ 500 W m^−2^, the operating temperature in the TPBR (average of the three temperature readings) remained relatively constant at approximately 36.2 °C (± 2.28 °C). Under varying light intensities, however, the operating temperature changed in accordance with light. That is, over a period of 24 h, the temperature ranged from about 17–18 °C during the dark periods to about 38–39 °C during the illuminated periods (Fig. 6). To investigate the individual effects of the simulated diurnal light cycle without the influence of varying temperature and circulation in the TPBR, the hydrogen production behaviour of *R. palustris* was also investigated in a temperature-controlled water bath reactor setup with constant mixing (Effect of Illumination Protocol on *R. palustris*).

## Discussion

In this study, the effect of diurnal light cycles on the growth and hydrogen productivity of *R. palustris* was investigated. Furthermore, the operation of a TPBR system was investigated under simulated diurnal light cycles, with the ultimate goal of the TPBR being to reduce operating costs associated with photofermentation systems. The long-term view for the TPBR is not only to produce hydrogen, but also to produce hydrogen from waste while being entirely independent of external energy inputs. The main operating costs associated with photofermentation systems are illumination, mixing and nutrients/substrate. Under outdoor conditions, using a passive cooling strategy like cooling fins, two of these costs can be eliminated when using the TPBR, i.e. mixing and illumination. To eliminate or at least reduce the cost of the third factor, waste streams rich in organic carbons could be utilised as substrate. Large volumes of glycerol are currently being produced in the biodiesel industry owing to significant biodiesel demand. As a result, the price of glycerol has fallen over the past few years (~ $0.30/L in 2012 to $0.10/L in 2019), which has caused it to gain attention in terms of waste-to-valuable products (Nomanbhay et al. 2018; Pott et al. 2014; Raman et al. 2019; Sabourin-Pruvost and Hallenbeck 2009). Several studies have reported the use of glycerol as carbon substrate for photofermentative hydrogen production by *R. palustris* (Bosman et al. 2022, 2023; du Toit and Pott 2021; Ghosh et al. 2012; Pott et al. 2013; Ross and Pott 2022; Sabourin-Pruvost and Hallenbeck 2009; Xiao 2017) and have demonstrated hydrogen production rates of up to ~ 32 mL g_CDW_^−1^ h^−1^ (Pott et al. 2013; Sabourin-Pruvost and Hallenbeck 2009), which compare well with production rates reported using more conventional carbon substrates—~ 15–40 mL g_CDW_^−1^ h^−1^ (Barbosa et al. 2001; Ghosh et al. 2012; Pott et al. 2013; Sabourin-Pruvost and Hallenbeck 2009; Vincenzini et al. 1982; Wang et al. 2010; Wu et al. 2010). Additionally, near-stoichiometric conversion of glycerol to hydrogen (close to 90%) have also been reported by Pott et al. (2013), collectively suggesting glycerol to be a suitable substrate for photofermentative hydrogen production by *R. palustris* and also offering a circular economics approach for green hydrogen production in synergy with waste management (Pott et al. 2018).

This study presents the first work done on the operation of a TPBR using *R. palustris* in combination with a ‘waste’ glycerol substrate under diurnal light cycles, implementing a novel light simulation system to closely mimic the light variation patterns of natural sunlight. To the authors’ knowledge, no work has been done on the effects of diurnal light cycles specifically on *R. palustris* and additionally, no work has been done where natural sunlight was as closely mimicked as in this study, i.e. varying the light in a smooth ramping up and down manner, like natural sunlight would vary, rather than step-wise variations in light intensity.

Many studies have been conducted on the effect of light protocol on photosynthetic bacteria (Miyake et al. 1999; Uyar et al. 2007; Androga et al. 2011); however, little work has been done simulating the light intensity patterns of natural sunlight. The most prominent finding in this study was the demonstration of hydrogen production in the novel TPBR with *R. palustris* operating under simulated diurnal light cycles, representing an advancement in the drive towards outdoor operation of more cost-effective photofermentative hydrogen production systems. With regards to the TPBR system being operated under outdoor conditions, natural sunlight would be sufficient for hydrogen production during the period of daylight, as hydrogen production was demonstrated in this study. However, the hydrogen production rates achieved in the TPBR using *R. palustris* were lower than the literature reported 0.09 mol m^−3^ h^−1^ for the purple bacterium *Rhodobacter sphaeroides* under 12 h light/12 h dark cycles in a glass column PBR (400 mL) (Eroǧlu et al. 2010). As a result, green energy would be required to provide artificial illumination to the system during the period outside of natural daylight, i.e. night-time, to further enhance hydrogen productivity.

Higher hydrogen production rates were achieved under continuous illumination as compared to cyclic illumination which were to be expected, as hydrogen production ceased over night during the dark periods and only resumed again in the presence of light (Fig. 4). In a study by Uyar et al. it was found that hydrogen production was substantially higher under continuous illumination (Uyar et al. 2007), while another study found the hydrogen production in the first 60 h to be higher under continuous illumination as compared to under 12 h light/12 h dark cycles, but with no discernible difference in volume of hydrogen produced at the end of the experimental run at ~ 132 h (Eroǧlu et al. 2010).

Cessation of hydrogen production during dark periods was also observed in two other studies on *Rhodobacter sphaeroides* (Miyake et al. 1999; Uyar et al. 2007), which consequently reduced hydrogen production and yield—in this study, under both varying temperatures (temperature in the TPBR varying with cyclic light intensity) as well as constant temperatures (temperature in test-tube reactors maintained at 35 °C with cyclic light). As the hydrogen production metabolism of *R. palustris* is both temperature and light-dependent (du Toit and Pott 2021; Ross and Pott 2022), hydrogen production was exhibited only during a small 6-h window (10:00–16:00) within the 24-h diurnal light cycle, in which temperature was within the range of 30–42 °C (du Toit and Pott 2021) and light intensity was above the lower physiological bound of approximately 70 W m^−2^ (Ross and Pott 2022), as indicated with the shaded area in Fig. 6. The low and high temperatures observed in the TPBR under cyclic illumination were due to the variation in light intensity—since the cyclic light intensity ramped up to about 900 W m^−2^ as compared to the lower light intensity of 500 W m^−2^ under continuous illumination, the maximum temperatures under cyclic illumination were slightly higher than that under continuous illumination. The higher light intensity caused the working fluid inside the reactor to be warmer and also allowed for increased absorption of light energy by the microorganisms.

The lower hydrogen productivity under cyclic illumination as compared to under continuous illumination can also be attributed to light intensity (Carlozzi 2009; Carlozzi et al. 2010; Ross and Pott 2022). The average light intensity of the diurnal light cycles over a period of 24 h was determined to be approximately 145 W m^−2^, which is relatively low for photofermentative hydrogen production by *R. palustris* (Carlozzi 2009; Carlozzi et al. 2010; Ross and Pott 2022), and also substantially lower than the continuous illumination light intensity that was maintained at 500 W m^−2^. Due to the reduced average light intensity and cessation during dark periods within the 24-h diurnal light cycles, an artificial light source should be considered for night-time operation. As the thermosiphon effect ceases in the absence of light/heat, the addition of artificial illumination is necessary for the TPBR to continue to passively circulate biomass, maintain biomass in suspension and of course also for photofermentative hydrogen production for the entire 24 h under outdoor conditions. To minimize the environmental burden of artificial illumination, a sustainable option would have to be implemented.

Biomass concentration continued to increase during dark periods—this could be attributed to several factors such as accumulation of storage products, i.e. PHB granules and/or bacterial cell growth—a phenomenon also observed by Eroǧlu et al. (2010). In the present study, PHB granules were observed for both continuous as well as cyclic illumination—these granules were similar to that identified and quantified in *R. palustris* TIE-1 in a study by Ranaivoarisoa et al*.* (2019). PHB granules are typically produced as a result of nutrient-starvation, i.e. nitrogen-starvation and/or unfavourable conditions (McKinlay and Harwood 2011; Chen et al. 2012; Wu et al. 2012). As mentioned, both temperature as well as circulation efficiency in the TPBR are light-dependent—both temperature and biomass circulation vary in accordance with light intensity and furthermore, circulation ceases in the absence of light. Therefore, the production of PHB was suspected to be due to a combination of reduced mixing and consequent nutrient transfer (McKinlay and Harwood 2011; Chen et al. 2012; Wu et al. 2012), as well as varying light intensities [“light-induced redox stress” (Alloul et al. 2022)] and temperature.

As shown in Fig. 6, the increase in temperature after the dark period (approximately 19:00 to 06:20) coincides with the increase in light intensity, i.e. temperature and light intensity start to increase at the same time. Little work has been conducted on the circadian rhythms of *R. palustris*, however, a study by Ma et al. (2016) suggested a time-keeping mechanism whereby *R. palustris* possesses an ability to maintain a periodicity to some extent without necessarily having the characteristic circadian rhythm. The organism does not have the ability to sustain rhythmicity in constant conditions (generally not found in the natural environment), but expresses a time-keeping mechanism based on adaptive properties to enhance fitness in rhythmic environments (natural environment) (Ma et al. 2016). In this specific study by Ma et al. *R. palustris* started to anticipate the light cycle (12 h light/12 h dark), by resuming metabolic activities (specifically nitrogen fixation) a short period before being exposed to the light (Ma et al. 2016). At higher biomass concentrations, should this have been the case, it is possible that the initial increase in temperature would have shifted slightly to the left with the organisms presumably emitting metabolic heat and increasing the operating temperature before being exposed to the light in the morning. However, because the system in this study was operated at very low biomass concentrations the contribution of metabolic heat would have been comparably less than the contribution of the light sources and therefore likely not measurable, providing no distinctive evidence of the organisms anticipating or not anticipating the light and exhibiting a 24-h time-keeping mechanism. Thus, the possibility of *R. palustris* possessing a time-keeping mechanism cannot be discarded and requires further investigation from a physiological perspective. With regards to the hydrogen production life cycle of *R. palustris* in a TPBR, operating under diurnal light cycles did seem to extend the period of production presumably due to the bacterial cells not being overexerted; however, at the cost of substantially lower production rates and volumes. This finding corroborates the term “proto-circadian” (Ma et al. 2016), as the extended metabolically active periods of the microbial cells suggest there is some sort of fitness/survival advantage, though not necessarily associated with the characteristic circadian rhythm. These findings provide valuable insight into the outdoor operation of not only the TPBR system, but also to other photofermentative hydrogen production systems operating with photosynthetic bacteria.

In conclusion, this study provides valuable knowledge and insight into the working of the TPBR system under simulated outdoor conditions. Furthermore, it also provides insight into the photofermentative hydrogen production of the purple non-sulphur bacteria *R. palustris*. Collectively, this study adds to the advancement of photobioreactors and outdoor operation, and therefore also to the potential industrialization of photofermentative hydrogen production systems.

## Data Availability

The datasets generated and/or analysed during the current study are available from the corresponding author on reasonable request.

## Abbreviations

*R. palustris *: *Rhodopseudomonas palustris*
PNSB: Purple non-sulphur bacteria
TPBR: Thermosiphon photobioreactor
PBR: Photobioreactor
CDW: Cell dry weight
OD: Optical density
NTP: Standard temperature and pressure
PHB: Poly-hydroxybutyrate
STEM: Scanning transmission electron microscopy
CAF: Central analytical facilities
